# Neighborhood Characteristics and Cancer Survivorship: An Overview of the Current Literature on Neighborhood Landscapes and Cancer Care

**DOI:** 10.3390/ijerph18137192

**Published:** 2021-07-05

**Authors:** Sima Namin, Yuhong Zhou, Joan Neuner, Kirsten Beyer

**Affiliations:** 1 Institute for Health & Equity, Medical College of Wisconsin, Milwaukee, WI 53226, USA; yuzhou@mcw.edu (Y.Z.); kbeyer@mcw.edu (K.B.); 2 General Internal Medicine, Medical College of Wisconsin, Milwaukee, WI 53226, USA; jneuner@mcw.edu

**Keywords:** cancer survivorship, neighborhood characteristics, health promotion, LEED-ND

## Abstract

There is a growing literature on the association between neighborhood contexts and cancer survivorship. To understand the current trends and the gaps in the literature, we aimed to answer the following questions: To what degree, and how, has cancer survivorship research accounted for neighborhood-level effects? What neighborhood metrics have been used to operationalize neighborhood factors? To what degree do the neighborhood level metrics considered in cancer research reflect neighborhood development as identified in the Leadership for Energy and Environmental Design for Neighborhood Development (LEED-ND) guidelines? We first conducted a review guided by PRISMA extension for scoping review of the extant literature on neighborhood effects and cancer survivorship outcomes from January 2000 to January 2021. Second, we categorized the studied neighborhood metrics under six main themes. Third, we assessed the findings based on the LEED-ND guidelines to identify the most relevant neighborhood metrics in association with areas of focus in cancer survivorship care and research. The search results were scoped to 291 relevant peer-reviewed journal articles. Results show that survivorship disparities, primary care, and weight management are the main themes in the literature. Additionally, most articles rely on neighborhood SES as the primary (or only) examined neighborhood level metric. We argue that the expansion of interdisciplinary research to include neighborhood metrics endorsed by current paradigms in salutogenic urban design can enhance the understanding of the role of socioecological context in survivorship care and outcomes.

## 1. Introduction

Cancer is the second leading cause of death globally, accounting for 9.6 million deaths and 18.1 million new cases in 2018 [1]. With advancements in treatment and early detection, the number of cancer survivors is increasing [2]. In 2018 the number of cancer survivors diagnosed within the past 5 years was estimated at around 43.8 million globally [3]. The number of cancer survivors in the U.S. is also rapidly growing with a current estimate of 16.9 million cancer survivors that is expected to rise to 22.1 million by 2030 [4]. As the number of cancer survivors increases, it poses challenges to health care systems [3]. Moreover, a large body of literature shows inequities in survivorship outcomes and experiences in minority and disadvantaged populations [5,6]. Therefore, reducing disparities across the survivorship continuum is a priority for health promotion and clinical care.

The field of survivorship research has identified six key elements of survivorship as (1) surveillance for recurrence and screening for second cancers [7]; (2) issues related to long-term and late effects of cancer treatment [8]; (3) health promotion through behavioral changes including weight management and physical activity [9], healthy diet [10], smoking cessation and reduced alcohol consumption [11,12]; (4) psychological well-being [13]; (5) survivorship needs in special populations [14]; and (6) empowering survivors to advocate for their health care needs [15]. The association between neighborhood/social contexts and these key elements in cancer survivorship is widely established. For example, researchers have studied the relationship between screening adherence and neighborhood-level socioeconomic predictors [16]; many studies have highlighted neighborhood influences on physical activity and cancer outcomes [17]; neighborhood food environment and cancer outcomes [18]; the effects of neighborhood socioeconomic context on smoking cessation and alcohol consumption and overall survival [19]. There is also a growing literature on the effects of neighborhood factors on mental well-being among cancer survivors [20]. Furthermore, the effects of neighborhood social networks and social isolation have been studied in relation to preventive care utilization and cancer outcomes [21,22]. There is also abundant literature on access to green space and mental health among cancer survivors [23].

Although there is a growing literature on social determinants of health and behavioral factors influenced by built environment, to our knowledge there are no reviews focused on the question of cancer survivorship in the context of neighborhood effects and utilized metrics. In this study, we used a scoping review process, a knowledge synthesis approach focused on mapping the key concepts and the states of knowledge [24,25], to answer three research questions:

RQ1: To what degree, and how, has cancer survivorship research accounted for neighborhood-level effects?

RQ2: What neighborhood metrics have been used to operationalize neighborhood factors?

RQ3: To what degree do the neighborhood level metrics considered in cancer research reflect neighborhood development, as identified in the Leadership for Energy and Environmental Design for Neighborhood Development (LEED-ND) guidelines?

To answer these questions, we first conducted a review of the extant literature on neighborhood effects/metrics and cancer survivorship care and outcomes. We then assessed the findings based on one of the current guidelines in community health and neighborhood development (Leadership for Energy and Environmental Design guidelines for Neighborhood Development (LEED-ND)) [26] to identify the overlaps and gaps between these findings and the six areas of focus in cancer survivorship research.

## 2. Materials and Methods

Given the broad nature of our research questions, we employed PRISMA extension for scoping review [27] to extract the main themes and trends of this wide literature. The aim of scoping analysis is to map the key concepts in a research area and the main evidence available, which is particularly useful in emerging, multifaceted, and multidisciplinary issues [28]. A scoping analysis consists of an iterative six-stage process: (1) identifying the research question, (2) identifying relevant studies, (3) study selection, (4) charting the data, (5) collating, summarizing and reporting the results, and (6) an optional consultation exercise [25].

A discussion on the advantages and limitations of scoping analysis, in general, can be found elsewhere [29]. Employing a scoping analysis, we focus on the conceptual framework of the existing literature. We identify the main themes, subthemes, and utilized variables in the studied articles. In the following sections, we go through the steps of scoping analysis in detail.

### 2.1. Search Strategy and Identification of Relevant Studies

We first identified three main scientific fields on which to focus our literature search: medical science, interdisciplinary sciences, and social sciences. We then identified 2 to 3 databases to search for each field: medical science (PubMed, CINAHL), interdisciplinary sciences (Scopus, Web of Science, a metasearch engine which access to several databases), and social science (ERIC, JSTOR and PsychINFO). We searched for peer-reviewed journal articles published in English between 1 January 2000 and 1 January 2021. We searched these 7 academic databases using a very specific set of terms: (((neighborhood) OR (neighborhood AND characteristics) OR (built AND environment) OR (neighborhood AND design)) AND ((cancer AND survivorship) OR (cancer AND care) OR (cancer AND survivor))).

### 2.2. Inclusion Criteria

To be included in the review, each article had to meet four criteria. First, it had to be an original research paper, so papers or reports on protocols of ongoing/prospective research were not included. In addition, we did not include gray literature in our review (e.g., dissertations and theses, LexisNexis Academic, NIH RePORTer, American Cancer Society). We compiled the review papers in a separate sheet and scanned them for additional relevant references. Second, the paper must examine survival; articles focused only on access to initial treatment, such as chemo or surgery, were not included. We defined cancer survivorship from the completion of primary treatment to the end of life [30]. Third, the article’s analyses had to include one or more neighborhood-level variable; therefore, studies that only reported on individual-level factors were not included. Fourth, studies had to include neighborhood or local-level characteristics and, therefore, studies with only county-level variables and international comparisons of survival outcomes were not included. Both qualitative and quantitative articles were included if they examined neighborhood metrics in association with cancer survivorship care or outcomes. Supplementary Table S1 lists our inclusion and exclusion criteria.

### 2.3. Data Processing and Extraction from Included Studies

After executing the search strategy, we first scanned the titles to exclude any articles that were clearly outside of the scope of our analysis. Second, we assessed the abstracts of potentially relevant articles and made another set of exclusions. We then assessed the full text of the remaining abstracts based on the inclusion criteria and determined a final set of articles. We exported the eligible articles into a spreadsheet for data extraction. One author reviewed the articles through each stage of the assessment; a second author assessed a random subset of the results. The final dataset was compiled after a consensus was reached regarding the inclusion/exclusion decisions. We recorded the following data in a spreadsheet: title, author(s), publication year, neighborhood metrics, location, level of geography, data sources, cancer type, method, main findings. The extracted data were double-checked by the authors to ensure accuracy. Each article’s bibliographic information was imported into Mendeley [31] for data management and referencing purposes. We used Microsoft Excel [32] and Raw Graphs [33] for visualization and analysis.

Supplementary Table S2 in the Supplementary Materials contains the full list of included articles. The strength of the evidence for quantitative studies was ranked as low, medium or high by considering sample size, the use of a neighborhood measure, whether neighborhood metrics were the primary predictor, study generalizability, and use of appropriate covariate [34]. Qualitative studies were assessed based on argument cohesiveness and understanding of neighborhood variables associated with cancer survivorship care and outcomes.

### 2.4. Collating, Summarizing and Reporting the Findings

We summarized the results based on the main theme and primary variable in each study. We used an alluvial diagram to show the distributions across different themes and their corresponding variable to highlight broader trends in the current body of literature. We developed a summary chart to classify the main themes. We chose this thematic classification to provide an overview of the neighborhood-level metrics in relation to the main areas of focus in cancer survivorship research.

### 2.5. Conceptualizing the Findings

We used the LEED for Neighborhood Development (LEED-ND) metrics, which was developed in collaboration between the United States Green Building Council, the Congress for the New Urbanism, and the Natural Resources Defense Council to organize and to conceptualize the findings on neighborhood level variables. We use the LEED_ND framework as a reference for an interdisciplinary approach in understanding neighborhood context and community health because promoting community health, well-being, and quality of life is one of the overarching goals of the LEED_ND rating system. Moreover, in 2007, an expert review panel, convened by the CDC [35], concluded that several of the LEED_ND metrics could contribute to promoting community and individual health through (1) reducing the risk of obesity, heart disease, and hypertension; (2) reducing air pollution and respiratory diseases; (3) increasing social connection and sense of community; (4) improving mental health; (5) encouraging healthier diets. To conceptualize the findings, we compared the neighborhood variables and the themes that emerged from the examined articles to the relevant metrics in the LEED-ND system. We later rely on this comparison to provide a synthesis of the findings.

## 3. Results

**Figure 1:**
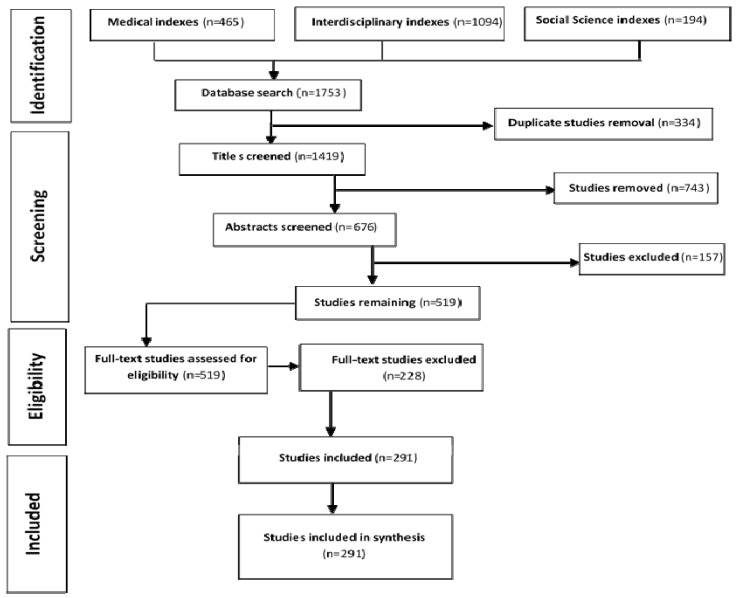
Review process of the search results by search database guided by PRISMA. Medical indexes: PubMed, CINAHL. Interdisciplinary indexes: Scopus, Web of science. Social science indexes: ERIC, JSTOR, and PsychINFO.

Figure 1 shows the process of identifying relevant articles. The search captured 1753 results in total, which was reduced to 1419 after duplicates were removed. Title scans resulted in a total of 676 articles that were later reviewed by abstract. The abstract review resulted in 519 articles from all the sources. After full-text review, we identified a total of 291 articles.

**Figure 2:**
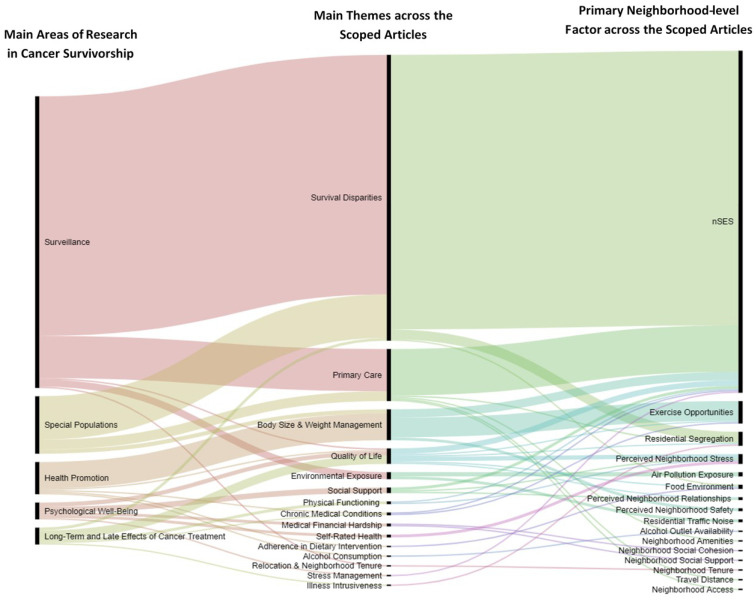
Main themes and primary neighborhood factors across the scoped articles.

Almost 42% of the articles were published after 2015, indicating growing attention to neighborhood effects on cancer survival care and outcomes. Additionally, 31% of the articles were on breast cancer, followed by 27% on more than one cancer site and 12% on colorectal cancer. Figure 2 provides a visualization of the main themes and primary neighborhood-level variables across the scoped articles. Overall, 68.7% of all the studies were focused on survival disparities, 12.3% on issues related to primary care, 7.5% on weight management among cancer survivors, and 3.7% on quality of life. Many neighborhood-level variables can affect cancer outcomes, for example, through affordability and access for adhering to healthy diets and opportunities for physical activity. Survival disparities and primary care issues have been studied mostly in relation to nSES and residential segregation, whereas for body size management and quality of life, neighborhood design variables related to exercise opportunities, neighborhood stress and social contexts have been studied.

**Figure 3:**
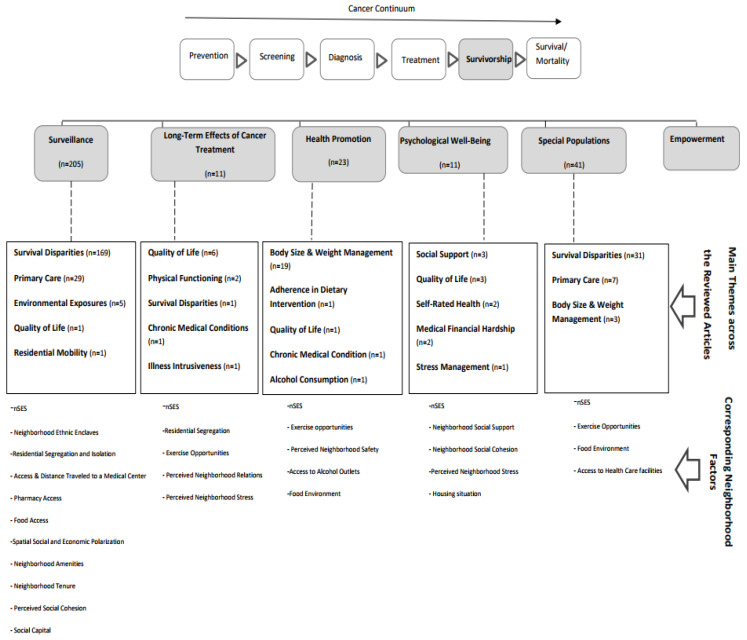
Main themes and the corresponding neighborhood metrics in the scoped articles.

Figure 3 provides a visual representation of the main themes and the complete list of the corresponding neighborhood metrics employed in the scoped articles. The main themes under surveillance were survival disparities (*n* = 169), primary care (*n* = 29), environmental exposure (*n* = 5), quality of life (*n* = 1), residential mobility (*n* = 1). The neighborhood variables utilized to study these themes were nSES, racial and ethnic composition and segregation, medical care access (pharmacy and care delivery facilities), food access and neighborhood amenities, and neighborhood social context and neighborhood tenure. For the long-term effects of cancer treatment, the main themes were quality of life (*n* = 6), physical functioning (*n* = 2), survival disparities (*n* = 1), chronic conditions (*n* = 1), and illness intrusiveness (*n* = 1). Neighborhood variables utilized in this category were nSES, residential segregation, exercise opportunities and perceived neighborhood relations and stress. In the health promotion category, the observed themes were body size and weight management (*n* = 19), diet adherence (*n* = 1), quality of life (*n* = 1), chronic medical conditions (*n* = 1), alcohol consumption (*n* = 1). The corresponding neighborhood variables in this category were nSES, exercise opportunities, neighborhood safety, alcohol outlets and food environment. The studied themes under psychological well-being were social support (*n* = 3), quality of life (*n* = 3), self-rated health (*n* = 2), medical financial hardship (*n* = 1) and stress management (*n* = 1). nSES, neighborhood social support and cohesion, perceived neighborhood stress and housing situation were the utilized neighborhood variables under this category. Lastly, for special populations, the studied themes were survival disparities (*n* = 31), primary care (*n* = 7), body size and weight management (*n* = 3), and neighborhood variables under this category were nSES, exercise opportunities, access to health care facilities, and food environment.

### 3.1. Main Areas of Cancer Survivorship Research and Utilized Neighborhood Metrics

#### 3.1.1. Surveillance

Among the scoped articles, survival disparities, primary care, environmental exposures, quality of life, and residential mobility were the main themes related to surveillance. Neighborhood measures utilized in these articles were: nSES, neighborhood ethnic enclaves [36], residential segregation [37], access and travel distance [38], pharmacy access [39], food access [18], social and economic polarization [40], neighborhood amenities [39], neighborhood tenure [41], perceived social cohesion [41,42], and social capital [43]. nSES was the most common metric and there is abundant literature that shows lower nSES exerts a deleterious effect on cancer outcomes which goes beyond the area supply of specific heath care types [44]. Most articles relied on nSES quantiles as one of the primary variables to analyze the survival outcomes and utlization of surveillance care and mostly found that survival advantage of living in higher nSES areas was significant [45,46]. Some studies also found that nSES is independently associated with an increased likelihood of receiving care [47]. Studies also found that ethnic enclave creates an survival advantage among Hispanic populations in California [48]. Residential segregation was the second most studied neighborhood metric under surveillance for analyzing survival disparities. Some of these articles found that residential racial composition had a significant negative effect on survival outcomes [49,50]. Other studies found lower hazards rate for breast cancer survival outcomes for more segregated areas [51]. One study also showed that access and travel distance to primary care is significantly related to survival outcomes [38]. Another study found that neighborhood rating based on perceived neighborhood characteristics was associated with access to prescription opioids [39]. One article showed that social and economic polarization was associated with receipt of a Survivorship Care Plan (SCP) [40]. Another study found that the odds of neighborhood relocation among cancer survivors were negatively associated with nSES and perceived neighborhood social cohesion [41]. Studies also suggest that neighborhood social capital in barrios might explain the positive effects on survivorship outcomes among Mexican American women breast cancer survivors [43].

#### 3.1.2. Long-Term Effects of Cancer Treatment

Across the scoped articles, quality of life, physical functioning, survival disparities, chronic medical conditions, and illness intrusiveness were the main studied themes related to long-term effects of cancer treatment. Neighborhood measures to study these themes were: nSES, residential segregation [52], exercise opportunities (e.g., active transportation, walkability, neighborhood amenities) [53], perceived neighborhood relations [54], and perceived neighborhood stress [55]. Studies utilizing nSES found that there is a relationship between neighborhood deprivation and long terms effect of treatments and quality of life and physical functioning after cancer survival [56,57]. One study that utilized residential segregation showed that racial minorities living in segregated areas had higher odds of greater illness intrusion [52]. Additionally, neighborhood metrics that can affect exercise opportunities were also among the most studied themes. For example, one study found that living on streets with high-quality sidewalks was significantly associated with better emotional well-being and social functioning [53]. Perceived neighborhood relations and perceived neighborhood stress were also found to be associated with quality of life after cancer treatment [54,55].

#### 3.1.3. Health Promotion

Across the scoped articles, body size and weight management, adherence to diet, quality of life, chronic medical conditions, and availability of alcohol outlets were the main themes. Neighborhood measures were: nSES, exercise opportunities (e.g., neighborhood amenities including, parks, nature trails, green belts, recreational facilities, street connectivity and walkability) [58,59,60,61,62], food environment (e.g., farmers’ markets, convenience stores, liquor stores, fast-food restaurants, supermarkets, and restaurants) [63], perceived neighborhood safety [64,65], and access to alcohol outlets [66]. One article that looked at neighborhood exercise opportunities found that neighborhood amenities are a significant predictors of achieving sufficient levels of physical activity in cancer survivors [67]. However, one study showed that built environment variables were not associated with PA levels [68]. One article on social needs and health-related quality of life found that food insecurity is prevalent among cancer survivors (14.8%) and many neighborhood factors such as access to transportation and neighborhood safety affects patient’s decisions to forgo of their health care needs [63]. One study on improving health among breast cancer survivors found that living within 3 miles of alcohol outlets was significantly related to excessive alcohol consumption [66].

#### 3.1.4. Psychological Well-Being

Across the scoped articles, social support, quality of life, self-rated health, medical and financial hardships, and stress management were the studied themes. Among these reviewed articles, nSES, neighborhood social support [22], neighborhood social cohesion [42], perceived neighborhood stress [69], and housing situation were the neighborhood-level variables under the psychological well-being theme. One study found a strong association between social support and financial circumstances and one or more unmet needs among head and neck cancer survivors [22]. Regarding neighborhood social stress, one study found that socioecologic stress was the most important factor influencing physical and mental health-related quality of life [70]. Other studies found that neighborhood stress was significantly associated with poorer self-reported health [71]. Another study found that women who resided in high-foreclosure-risk (HFR) areas with higher perceived neighborhood conditions were more likely to report being in fair–poor health [72].

#### 3.1.5. Special Populations

Older cancer survivors and survivors of childhood cancers are considered special populations. These populations often experience more significant decline in functionality and long-term health conditions, making them vulnerable survivor populations [14]. Across the scoped articles, survival disparities, primary care, and body size and weight management were the main themes. Neighborhood factors to study these themes were: nSES, exercise opportunities [73,74], access to healthy food options [73], and access to health care facilities [44]. One study found that obese survivors of pediatrics cancers were more likely to live in neighborhoods with lower SES [73].

Studies on older cancer survivors also showed that living in high-poverty neighborhoods is associated with poor outcomes [75]. Another study found that education and language proficiency as measures of nSES were associated with higher mortality [76].

### 3.2. Overlaps between Neighborhood Metrics Utilized in the Scoped Articles and LEED-ND Metrics

Table 1 provides a summary of the 16 LEED-ND metrics that are relevant for healthy neighborhoods and the corresponding variation of these metrics across the scoped articles under the LEED categories of smart location and linkages and neighborhood pattern and design. Comparing the results with the utilized neighborhood metrics in the scoped articles (Table 1), we found some variation of 12 of them in the scoped articles. These measures included indicators of connectedness (intersection density); opportunities for physical activity (presence of bike paths, access to recreational facilities and civic spaces, walkable streets, street connectivity, presence of tree shade); compact development (number of housing units per square miles); diversity of uses (presence of mixed land uses); diversity of housing type (percentage of not single-family dwellings as a measure of diversity); local food production (food environment index).

## 4. Discussion

Our first question was: To what degree, and how, has cancer survivorship research accounted for neighborhood-level effects/metrics? To answer this question, we conducted a scoping review and provided a summary of the main themes and variables in the scoped articles (see Figure 2). We identified 15 themes that were studied in relation to neighborhood factors: survival disparities, primary care, body size and weight management, quality of life, environmental exposure, social support, physical functioning, chronic medical conditions, medical financial hardship, self-rated health, adherence in dietary interventions, alcohol consumption, relocation and neighborhood tenure, stress management, and illness intrusiveness. Our second question was: What neighborhood criteria/metrics are used to measure each area of focus in cancer survivorship? To answer these questions, we organized the identified themes under the umbrella on the six main areas of cancer survivorship research. We then listed the neighborhood variables that were utilized in studying each theme (see Figure 3). Our findings indicate that nSES, physical activity (exercise opportunities, walkability, and active transportation) and residential segregation are the most-studied neighborhood factors across these themes. To answer the third question: To what degree do the neighborhood level metrics considered in cancer research reflect neighborhood development as identified in the (LEED-ND) guidelines? We provided a comparison of the existing LEED-ND metrics related to healthy communities and their corresponding variant in the scoped articles (Please see Table 1). We identified 16 relevant themes in LEED-ND and, comparing the results with the utilized neighborhood metrics in the scoped articles, we found some variation of 12 of them in the scoped articles.

As the results of the scoped articles show despite considerable progress, until today the efforts for development of quality measures in cancer survivorship care and surveillance continue [84]. Moreover, persistent socioeconomic and racial disparities are translated into unequal access to ongoing quality medical care across different regions and disparate healthcare settings. Studies have found statistically significant interactions between neighborhood metrics and survival outcome though different pathways, including access to care [38], neighborhood food environment [18]. and housing stability [41]. Understanding these factors and their interaction will improve survival outcomes in vulnerable populations and help to identify effective intervention policies.

Moreover, research suggests that racial minorities living in areas with a higher percentage of racial minorities have higher odds of greater illness intrusion [52]. Other studies show that survivors living in neighborhoods with concentrated poverty report lower physical functioning [57]. Neighborhood built environment characteristics can also affect quality of life of cancer survivors through emotional well-being and social functioning [53,85].

Additionally, neighborhood environments affect many individual behavioral choices through different pathways including exercise opportunities for higher physical activity and weight management [77,80] healthy food and diet [83] and reduced alcohol consumption [66] which are all associated with improved survival after cancer diagnosis. These contextual determinants of health behavior are important in understanding health behavior choices and planning effective survival care and behavioral interventions.

Furthermore, cancer survivors suffer a variety of psychological and emotional consequences throughout their lifetime that can directly affect quality of life [86]. There is abundant literature on nature and mindfulness-based stress reduction [87], positive psychological effects of physical activity among cancer survivors [86,88], and social support as an important determinant in survival outcomes in cancer patients [89]. The relationship between the built environment and perceived social support and its mediating effects on psychological distress has been previously studied [90]. Overall, the effects of neighborhood social and built environment factors on psychological wellbeing of cancer survivors remains understudied.

It should also be noted that older populations are disproportionately affected by cancer and its associated long-term effects [91]. The intersection of aging and cancer requires more complicated care plans and neighborhood factors can pose significant barriers for active aging among older cancer survivors. Survivors of childhood cancer are also more likely to suffer from chronic conditions [92]. Expansion of utilized neighborhood level metrics can help in understanding of the interactions between neighborhood social and built environment and cancer care and outcomes among special populations.

Lastly, the theme of “empowerment” was not present in any of the scoped articles. Research shows that low level of empowerment is associated with low autonomy and social support with consequences for coping capacities in cancer survivors [93]. Empowering cancer survivors can contribute better control over patient health and health behavior [94]. Although empowerment is not addressed in any of the scoped articles; it should be noted that some of the components of empowerment can be mediated by social and neighborhood factors. For example, the literature suggests that social support is one of the important components of psychosocial empowerment in cancer survivors [95]. Moreover, given that the patient–physician relationship is an important catalyst in the patient empowerment process, disparities in quality primary care can be translated to less empowerment in vulnerable populations.

Table 1 provided a summary of LEED-ND metrics that are relevant for healthy neighborhoods and a comparison with utilized neighborhood variables across the scoped articles. The results showed that, out of 16 metrics in LEED-ND that are directly or indirectly related to human health, some variation of 12 of them were found in the scoped articles. These measures included indicators of connectedness; opportunities for physical activity; compact development; diversity of uses; diversity of housing type); and local food production. However, studies that utilize many of these measures are all from California, probably due to the availability of the California neighborhood atlas [96]. Overall, many of these measures, such as connected neighborhoods, street connectivity, continuous sidewalks, active transportation, local food environment, and access to green space and ecosystem services are understudied. Since cancer survivors, particularly special populations (older survivors and childhood cancer survivors), tend to be less active than the general population, neighborhood environment plays an important role in adherence to physical activity guidelines [74]. Therefore, LEED-ND measures for examining access, connectedness, walkability, and active transportation help in examining neighborhood effects in cancer outcome. Moreover, these factors are intertwined with other prerequisites of healthy behavior, such as food environment. Additionally, among the unutilized measures are housing diversity and affordable housing measures, neighborhood food production and gardening and share of green infrastructures to impervious surfaces. As the results of this review showed (Table 1 and Figure 3), residential segregation is one of the most studied neighborhood variables across the scoped articles. Since residential segregation is a complex socioeconomic indicator, inclusion of housing diversity and affordability measures is important in understanding the role of residential environment in cancer outcomes. Neighborhood food production is another important variable with implications for food environment and neighborhood social environment. Green infrastructure is also important in assessing the neighborhood environment in terms of access to ecosystem services that can potentially reduce exposure to respiratory hazards and promote physical activity. Incorporating these metrics can inform built environment interventions and improve health monitoring for cancer survivors. Moreover, different credit levels defined by LEED-ND for each of these variables can be replicated at different levels (e.g., block group, block level) for creating a quantified scale that can be incorporated into statistical models examining the relationship between neighborhood contexts and cancer survivorship outcomes.

## 5. Limitations

Our review has several limitations. First, only articles published in English are included and, therefore, this review excludes published works in other languages. Second, our search strategy was limited to seven databases and therefore our results do not reflect the whole scope of the field. However, to our knowledge, no other similar review on this topic has been done, thus, we believe that this review provides a useful synthesis of the literature on neighborhood-level variables in cancer survival research. Third, we did not conduct separate independent reviews that are common in scoping analysis [97] and relied on checks during the data processing to increase the efficiency and speed of the process. Fourth, although LEED ND has very specific guidelines that define different levels of credit and achievement for satisfying neighborhood measures, in our comparison we did not adhere to the full measures and instead any variation of the measures of LEED ND in the scoped articles were considered satisfactory for metric inclusion. Lastly, given the nature of the synthesis we avoided rigid classification of themes (Figure 3) as we observed occasional overlap between themes, such as quality of life.

## 6. Conclusions

Previous work shows the effect of built environment across the cancer continuum. Our results show that survivor disparities, primary care, weight management and quality of life are the main themes in the literature. The most utilized neighborhood measures were nSES, exercise opportunities and residential segregation. This review also found that neighborhood metrics utilized in cancer survivorship research largely overlap with LEED-ND metrics (Table 1). Many of these metrics have been utilized to explore disparities in cancer survivorship, neighborhood effects on health promotion and quality of life, as well as psychological well-being. (Figure 3). Therefore, creating an open source local/regional neighborhood atlas to cover LEED-ND metrics for small geographies, particularly for the category of neighborhood pattern and design, will likely encourage more research on the relationship between neighborhood context and cancer survivorship outcomes. Additionally, many of these neighborhood metrics can be studied in relation to adherence to health promotion intervention and trials for cancer survivors. Evaluation of outcome disparities using these metrics can also support cancer prevention efforts by neighborhood design interventions. Given that many of these measures are widely used in urban planning, design, and urban ecology, interdisciplinary collaboration will accelerate knowledge about the impact of these variables on survivorship.

## Figures and Tables

**Table 1:** LEED-ND Metrics for Healthy Neighborhoods [45,50] and the corresponding measures in the scoped articles.

Area	LEED-ND Metrics	Metrics in the Scoped Articles
SLL **Smart Location and Linkages**	**Smart Location** (access to public transportation and walkability). - At least 35 intersections per km2. - At least 50% of units in ¼ mile walking distance of transit. - Minimum 60 weekday by transit service. **Access to quality transit** (provide transportation choices and reducing motor vehicle use). - Within a 1/4-mile walking distance of at least one bus or streetcar stop, or within a 1/2-mile walking distance of at least one bus rapid transit stop, light or heavy rail station, commuter rail station. - Measures for minimum daily transit service for projects with multiple transit types. **Bicycle Network** (to promote bicycling). - Within 1/4-mile bicycling distance of an existing bicycle network, that connects to mixed use destinations. - At least 3 continuous miles. **Housing and Job Proximity** (providing opportunities for shorter vehicle trips and/or use of alternate modes of transportation). - Within a 1/2-mile walking distance of existing full-time equivalent job.	**Smart Location** - Gamma index as a measure of connectivity [77,78]; Intersection density [61,79] **Bicycle Network** - Presence of bike paths in public parks [61,79]
NPD **Neighborhood Pattern and Design**	**Compact Development** (promote livability, transportation efficiency, and walkability). - 12 or more dwelling buildable land units per acre. **Connected and Open Community** (to improve public health by encouraging daily physical activity). - At least 90–140 intersections per mi2. **Diversity of Uses** (promote community livability, transportation efficiency, and walkability). - At least 4–7 diverse uses within 1/4 mile. **Diversity of Housing Types** (integrating a wide range of economic levels and age groups to live within a community). - Simpson Diversity Index score greater than 0.5. **Affordable Rental and For-Sale Housing.** - Proportion of rental and/or for-sale dwelling units priced for households earning less than the area median income (AMI). **Reduced Parking Footprint** (to increase the pedestrian orientation and to minimize the adverse environmental effects). - No more than 20% of the total neighborhood footprint area for all new off-street surface parking facilities. **Walkable Streets** (promote public health through increased physical activity). - Continuous sidewalks or equivalent provisions for walking. - Percentage length with speed for safe pedestrian and bicycle travel. - Criteria for façades and entries of buildings in circulation network. **Tree-Lined and Shaded Streetscapes** (to encourage walking and bicycling). - Provide trees at intervals of no more than 50 feet along at least 60% of the neighborhood total existing blocks. **Street Network** (increase connectivity, promote multimodal transportation, and promote public health through increased physical activity). - Pedestrian or bicycle through-connections for 50% cul-de-sacs. - Street network grid density within ¼ mile radius. **Access to civic and Public Spaces** (to provide a variety of open spaces close to work and home to encourage walking, physical activity, and time spent outdoors). - Within a 1/4-mile walk of at least one civic and passive use space. **Access to Recreation Facilities.** - Access and proximity to publicly accessible indoor recreational facility within ½ mile walking. **Local Food Production** (promote community-based and local food production and increasing direct access to foods). - Proximity to farmer’s market. - Criteria for minimum neighborhood garden space. - Community supported agriculture within 150 miles.	**Compact Development** - Number of housing units per square mile [53] **Connected and Open Community** - Intersection density [61,79] **Diversity of Uses** - Presence of mixed-use on street segment [53]; total businesses within a one-mile pedestrian network distance [78] **Diversity of Housing Types** - Mixed housing [53]; percentage of total housing units that are not single-family dwellings [78,80] **Walkable Streets** - Walkability scale [81]; Buffer-level walkability [68]; per grid unit [82]; walkability score [74] **Tree-Lined and Shaded Streetscapes** - Presence of tree shade [53] **Street Network** - Street network characteristics [61]; street connectivity [78] **Access to civic and Public Spaces** - Percentage of land area comprised of both total green and open spaces for recreation and Park density [61]; Access within an 800 m buffer [82] **Access to Recreation Facilities** - Number recreational lefts within a buffer [79,80] **Local Food Production** - Produce density [83]; Retail Food Environment Index [78,80]

## Supplementary Materials

The following are available online at https://www.mdpi.com/article/10.3390/ijerph18137192/s1, Table S1: Eligibility criteria for published studies on cancer survivorship and neighborhood context, Table S2: Full list of scoped articles.
